# Advances in Nanomedicine for Precision Insulin Delivery

**DOI:** 10.3390/ph17070945

**Published:** 2024-07-15

**Authors:** Alfredo Caturano, Roberto Nilo, Davide Nilo, Vincenzo Russo, Erica Santonastaso, Raffaele Galiero, Luca Rinaldi, Marcellino Monda, Celestino Sardu, Raffaele Marfella, Ferdinando Carlo Sasso

**Affiliations:** 1 Department of Advanced Medical and Surgical Sciences, University of Campania Luigi Vanvitelli, 80138 Naples, Italy; 2 Department of Experimental Medicine, University of Campania Luigi Vanvitelli, 80138 Naples, Italy; 3 Data Collection G-STeP Research Core Facility, Fondazione Policlinico Universitario A. Gemelli IRCCS, 00168 Roma, Italy; 4 Department of Biology, College of Science and Technology, Sbarro Institute for Cancer Research and Molecular Medicine, Temple University, Philadelphia, PA 19122, USA; 5 Division of Cardiology, Department of Medical Translational Sciences, University of Campania Luigi Vanvitelli, 80138 Naples, Italy; 6 Independent Researcher, 81024 Maddaloni, Italy; 7 Department of Medicine and Health Sciences “Vincenzo Tiberio”, Università degli Studi del Molise, 86100 Campobasso, Italy

**Keywords:** nanomedicine, insulin, precision insulin delivery, next-generation nanocarriers, targeted drug delivery

## Abstract

Diabetes mellitus, which comprises a group of metabolic disorders affecting carbohydrate metabolism, is characterized by improper glucose utilization and excessive production, leading to hyperglycemia. The global prevalence of diabetes is rising, with projections indicating it will affect 783.2 million people by 2045. Insulin treatment is crucial, especially for type 1 diabetes, due to the lack of β-cell function. Intensive insulin therapy, involving multiple daily injections or continuous subcutaneous insulin infusion, has proven effective in reducing microvascular complications but poses a higher risk of severe hypoglycemia. Recent advancements in insulin formulations and delivery methods, such as ultra-rapid-acting analogs and inhaled insulin, offer potential benefits in terms of reducing hypoglycemia and improving glycemic control. However, the traditional subcutaneous injection method has drawbacks, including patient compliance issues and associated complications. Nanomedicine presents innovative solutions to these challenges, offering promising avenues for overcoming current drug limitations, enhancing cellular uptake, and improving pharmacokinetics and pharmacodynamics. Various nanocarriers, including liposomes, chitosan, and PLGA, provide protection against enzymatic degradation, improving drug stability and controlled release. These nanocarriers offer unique advantages, ranging from enhanced bioavailability and sustained release to specific targeting capabilities. While oral insulin delivery is being explored for better patient adherence and cost-effectiveness, other nanomedicine-based methods also show promise in improving delivery efficiency and patient outcomes. Safety concerns, including potential toxicity and immunogenicity issues, must be addressed, with the FDA providing guidance for the safe development of nanotechnology-based products. Future directions in nanomedicine will focus on creating next-generation nanocarriers with precise targeting, real-time monitoring, and stimuli-responsive features to optimize diabetes treatment outcomes and patient safety. This review delves into the current state of nanomedicine for insulin delivery, examining various types of nanocarriers and their mechanisms of action, and discussing the challenges and future directions in developing safe and effective nanomedicine-based therapies for diabetes management.

## 1. Introduction

Diabetes mellitus comprises a group of metabolic disorders affecting carbohydrate metabolism. In this condition, glucose is both inadequately utilized as an energy source and excessively produced due to inappropriate gluconeogenesis and glycogenolysis, leading to hyperglycemia [1].

The prevalence of diabetes has surged to alarming levels, with global estimates indicating that 10.5% of individuals aged 20–79 (536.6 million people) are affected, a figure projected to rise to 12.2% (783.2 million) by 2045 [2]. Over the next two decades, this prevalence is expected to double, impacting over half a billion people, with more than 75% residing in low- and middle-income countries [3]. The increase seen in developing nations is attributed to the adoption of Western lifestyle habits, including sedentary behavior, physical inactivity, and a high-energy diet [4,5].

The diagnosis of diabetes involves demonstrating elevated concentrations of glucose in the venous plasma or an increased glycated hemoglobin (A1C) level in the blood. Conventionally, diabetes is classified into various clinical categories, including type 1 or type 2 diabetes, gestational diabetes mellitus, and other specific types arising from various causes, such as genetic factors, exocrine pancreatic disorders, and certain medications [1,6]. Type 1 diabetes mellitus (T1DM) is characterized by an absence or near-absence of β-cell function, necessitating insulin therapy for survival. Insulin treatment stands as a cornerstone for individuals grappling with type 1 diabetes, given the characteristic absence or near-absence of β-cell function in this population. Apart from addressing hyperglycemia, insulinopenia, a hallmark of type 1 diabetes, can give rise to additional metabolic disturbances such as hypertriglyceridemia, ketoacidosis, and tissue catabolism, posing life-threatening risks [7]. Beyond its traditional metabolic effects, insulin has also been proven to have positive cardiovascular effects, which are diminished during insulin resistance [8,9]. For many decades after the groundbreaking discovery of insulin, severe metabolic decompensation was predominantly averted through once- or twice-daily injections. However, in the last four decades, mounting evidence supports the adoption of more intensive insulin replacement strategies for individuals with type 1 diabetes [10].

The Diabetes Control and Complications Trial demonstrated the efficacy of intensive therapy involving multiple daily injections or continuous subcutaneous insulin infusion using short-acting (regular) and intermediate-acting (NPH) human insulins [11,12,13]. Intensive control, achieving lower A1C levels (7%), correlated with an approximately 50% reduction in microvascular complications over a 6-year treatment period. Despite its benefits, intensive therapy was associated with a higher incidence of severe hypoglycemia compared to conventional treatment [11,14,15]. Follow-up studies revealed fewer macrovascular and microvascular complications in the group that underwent intensive treatment, and the positive impact persisted over two decades beyond the active treatment phase of the study [11,12,13,16]. Expanding our focus to type 2 diabetes (T2DM), insulin therapy also plays a crucial role in its management. While, initially, lifestyle modifications and oral medications are commonly employed, the progressive nature of type 2 diabetes often necessitates the inclusion of insulin therapy. Basal insulin, mealtime insulin, and correction insulin strategies are similarly applied in type 2 diabetes, offering personalized approaches to optimize glycemic control. The choice of insulin regimen depends on individual patient characteristics, preferences, and treatment goals [17,18,19]. Similarly to T1DM, T2DM is linked to an increased risk of both vascular and non-vascular complications. In fact, T2DM elevates the risk of a range of cardiovascular disorders approximately twofold [20,21,22]. Furthermore, T2DM is associated with diverse non-vascular conditions such as cancer, infections, liver disease, and sensorial, mental and nervous system disorders [7,23,24,25,26,27,28,29,30].

Insulin replacement plans typically encompass basal insulin, mealtime insulin, and correction insulin [17]. Basal insulin options include NPH insulin, long-acting insulin analogs, and continuous delivery of rapid-acting insulin through an insulin pump. Basal insulin analogs exhibit a prolonged duration of action with more consistent plasma concentrations compared to NPH insulin, while rapid-acting analogs (RAA) offer a quicker onset and peak with a shorter duration of action than regular human insulin. In individuals with type 1 diabetes, treatment with analog insulins is associated with reduced hypoglycemia, weight gain, and lower A1C compared to injectable human insulins [31,32,33].

Recent advancements have introduced two injectable ultra-rapid-acting analog (URAA) insulin formulations designed to expedite absorption and provide heightened activity in the initial phase of their profile compared to other RAA [34,35]. Inhaled human insulin, featuring a rapid peak and shortened duration of action, presents an alternative option. These newer formulations exhibit potential advantages, including a reduced hypoglycemia risk, improved postprandial glucose control, and enhanced administration flexibility in relation to prandial intake compared to traditional RAA [36,37,38].

Subcutaneous insulin injections are effective. Despite being the current gold standard for diabetes management, these injections can lead to poor adherence to treatment plans, thereby compromising effective diabetes control, due to the high risk of severe hypoglycemia and the burdensome need for multiple daily injections [18,19,39]. This issue persists even with newer devices like microneedle delivery systems, which are simpler, more adaptable, and tend to be more acceptable to patients compared to traditional needle injections [40]. The necessity for novel approaches like nanomedicine becomes evident in addressing these challenges. Nanomedicine offers a promising alternative by enhancing medication targeting and stability, potentially reducing the frequency of administration and minimizing the risk of hypoglycemia. By improving drug delivery mechanisms, nanomedicine can significantly increase patient compliance and overall treatment efficacy, revolutionizing diabetes care and offering a more convenient and safer therapeutic option for patients. While inhaled insulin provides a non-invasive alternative, it may pose challenges related to pulmonary deposition and long-term safety [41]. Among these, oral insulin delivery holds significant promise due to its potential to improve patient adherence and quality of life. Despite its potential, oral insulin delivery faces substantial barriers, primarily due to the harsh gastrointestinal (GI) environment, which degrades insulin and impedes its absorption. Strategies to overcome these barriers include the development of advanced delivery systems, such as nanoparticles, which enhance insulin bioavailability and permeability across the intestinal mucosa [42]. Nanocarriers, such as liposomes, chitosan, and PLGA (Poly(lactic-co-glycolic) Acid), provide protection against enzymatic degradation, improving drug stability and controlled release. These nanocarriers offer unique advantages, ranging from enhanced bioavailability and sustained release to specific targeting capabilities [19,42].

Nanomedicine presents innovative solutions to the challenges of insulin delivery, offering promising avenues for overcoming current drug limitations, enhancing cellular uptake, and improving pharmacokinetics and pharmacodynamics [43]. This review delves into the current state of nanomedicine for insulin delivery, examining various types of nanocarriers and their mechanisms of action, and discussing the challenges and future directions in developing safe and effective nanomedicine-based therapies for diabetes management.

## 2. Challenges to Oral Insulin Administration and Nanomedicines

Insulin, a globular protein with a molecular weight of 5808 Daltons, consists of two chains, A (21 residues) and B (30 residues), linked together by disulfide bonds [44]. Currently, insulin is primarily administered through subcutaneous injections [45]. However, this delivery method is associated with several drawbacks, including lipohypertrophy, obesity, retinopathy, hypoglycemia, neuropathy, lipoatrophy, allergic reactions, and peripheral hyperinsulinemia [46,47]. The need for multiple insulin injections throughout the day can be burdensome for patients, leading to non-compliance. To address these challenges, there is a growing demand for controlled and prolonged release systems that reduce the injection frequency and enhance patient compliance. The Health Care Costs Institute reports a doubling of the cost of insulin for patients, emphasizing the urgent need for more patient-friendly alternatives. While subcutaneous injections are the current norm, oral delivery of insulin presents an attractive alternative due to the improved patient adherence and cost-effective manufacturing processes compared to injections [48].

### 2.1. Physiological Barriers in the Gastrointestinal Tract (GIT)

The harsh conditions of the gastrointestinal tract pose significant challenges to the oral absorption of peptide drugs. Factors such as the low pH, the presence of peptidases and proteases, and poor absorption through the intestinal epithelial layer hinder effective drug delivery [49,50].

#### 2.1.1. Gastric Acid Barrier

The stomach serves as one of the most formidable chemical barriers to insulin bioavailability. Gastric acid, which includes hydrochloric acid, is a fluid produced by parietal cells within the gastric glands of the stomach and used for digestion and protection from external potential infections. This secretion creates a highly acidic environment (pH 1–2) [51]. In such acidic conditions, free insulin is prone to degradation and proteolysis by the enzyme pepsin. Therefore, protecting insulin from the acidic and enzymatic environment is crucial for the effective oral delivery of insulin [52].

#### 2.1.2. Intestinal Mucosal Barrier

The intestine, the longest segment of the digestive tract, plays a critical role in both digestion and nutrient absorption, as well as in immune defense. The intestinal mucosal lining, also known as the intestinal barrier, functions to prevent pathogens from entering the bloodstream and other tissues [53]. The surface of the intestine is coated with mucus, which acts as a barrier between the luminal contents and the intestinal epithelium. This mucus is a gel-like network composed of highly glycosylated mucin molecules, which ensures the structural integrity and protective function of the intestinal barrier. Goblet cells are responsible for maintaining and renewing the mucus layer approximately every 4–5 h by secreting adhesion proteins and endophytes. The mucus selectively allows small molecules to permeate, while larger particles and pathogens are blocked, providing an anti-infective effect [54,55]. Hence, an effective oral insulin delivery system must traverse the mucus barrier to reach the intestinal epithelium.

**Figure 1:**
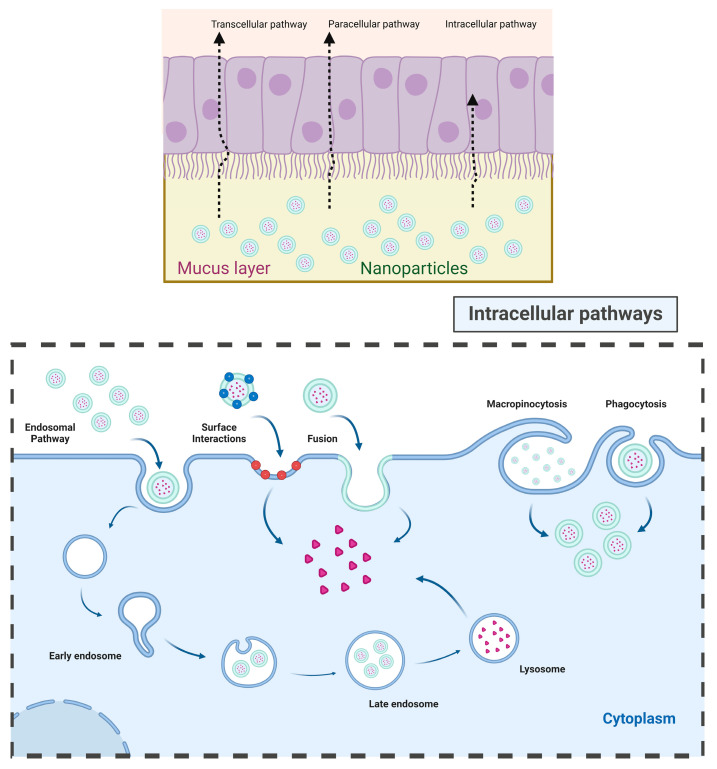
Barriers in the gastrointestinal tract to oral insulin delivery and potential pathways.

**Figure 2:**
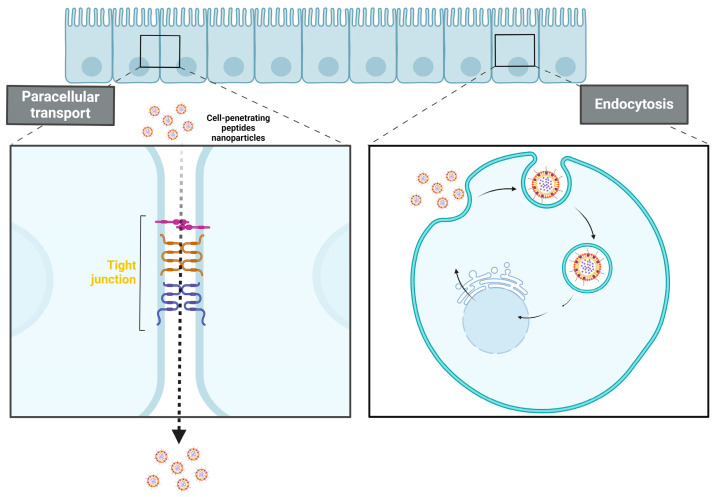
Mechanisms of the gastrointestinal barrier: gap junctions and intracellular pathways for oral insulin delivery.

Below the mucus layer, the intestine is lined with epithelial cells, which are the primary components of the intestinal mucosal barrier (Figure 1). For oral insulin to be effective, it must cross the intestinal epithelium via transcellular or paracellular pathways before it can enter the bloodstream and regulate blood glucose levels (Figure 2) [56]. The paracellular pathway, regulated by tight junctions between adjacent epithelial cells, is the preferred route for hydrophilic molecule translocation. However, most oral drugs are absorbed through the transcellular pathway, which involves processes such as intermembrane transport, adsorption, fusion, and endocytosis. The paracellular pathway is much more restrictive due to the tight junctions that create a sealing space of about 8–13 Å, which limits transport to hydrophilic molecules with molecular weights under 200 Da. In fact, the hydrophilic nature and large molecular size of peptide drugs further limit their oral absorption. One major obstacle to oral insulin delivery is the low bioavailability, often falling below 1%, attributed to enzymatic degradation in the gastrointestinal tract and inefficient absorption through intestinal epithelial cells [57]. Overcoming these barriers is crucial for the successful development of an effective and reliable oral insulin delivery system.

#### 2.1.3. Intestinal Wall Receptors and Delivery Systems

The intestinal wall hosts a variety of receptors, including vitamin B12 and folate receptors, and neonatal Fc receptors (FcRn). These receptors, located primarily on the apical surface of enterocytes, are crucial for facilitating the uptake of nutrients and can be strategically targeted to enhance the delivery and absorption of various therapeutic agents [58].

Vitamins are frequently used as ligands to enhance functional systems due to their safety, stability, and ease of modification. Among these, B12 and folic acid are the most extensively studied for oral targeted insulin delivery [59].

Vitamin B12 is a complex water-soluble molecule that includes a “corrin ring” and a nucleotide. It is absorbed orally after binding to haptocorrin, a salivary enzyme that protects and transports B12 to the small intestine [60]. In the intestine, B12 binds to an intrinsic factor to form a complex that interacts with receptors on enterocytes in the ileum. This mechanism has been used to improve the oral bioavailability of various poorly absorbed drugs, proteins, and peptides, including insulin [60]. In fact, B12-decorated dextran nanoparticles containing insulin significantly increased pharmacological availability (2.6-fold higher) compared to non-targeted nanoparticles [61]. By adjusting the parameters of the crosslinking and using an amino alkyl B12 derivative, researchers achieved a significant hypoglycemic effect and extended the antidiabetic effects, increasing insulin’s pharmacological availability up to 29.4% [62]. However, the potential applications of B12 are limited by its relatively slower uptake compared to other vitamins and the restricted availability of absorption sites, primarily in the distal ileum [63,64].

Folic acid (vitamin B9) is absorbed through a saturable, pH- and sodium ion-dependent pathway that is sensitive to metabolic inhibitors [65]. Unlike B12, folic acid receptors are abundant and enhance the uptake and transport of bioactive molecules across the gastrointestinal tract. Moreover, folate receptors exhibit a high affinity for folate and folate-conjugated compounds, making them effective targets for drug delivery via receptor-mediated endocytosis. Folate-decorated nanoparticles show great promise in enhancing the bioavailability of insulin. When insulin is encapsulated within these nanoparticles, they can specifically bind to folate receptors on the surface of intestinal epithelial cells. This binding facilitates receptor-mediated endocytosis, allowing the nanoparticles to cross the intestinal barrier and release insulin into the bloodstream. This targeted delivery not only protects insulin from enzymatic degradation and acidic conditions in the gastrointestinal tract but also enhances its absorption and bioavailability. By leveraging specific interactions with folate receptors, folate-decorated nanoparticles offer significant advancements in the oral delivery of insulin, potentially leading to better therapeutic outcomes and improved patient compliance [66,67,68,69,70].

The use of neonatal Fc receptors (FcRn) to develop intestinal targeted delivery systems has gained momentum [71]. These receptors, available from the neonatal stage into adulthood in the human intestine, bind immunoglobulin G (IgG) and albumin, protecting them from intracellular enzymatic breakdown [72]. By using the Fc portion of IgG or albumin as a targeting ligand, drugs/nanocarriers can be transcytosed by endosomes and released in the extracellular space at a physiological pH [73,74]. FcRn-targeted poly(lactic acid)–poly(ethylene glycol) (PEG-PLA) block copolymer-based nanoparticles, with the Fc portion of IgG linked to their surface and loaded with insulin, demonstrating a hypoglycemic effect in vivo [75]. Finally, when insulin was loaded into nanoparticles functionalized with the Fc fragment of IgG as a targeting ligand to the FcRn, its permeation across an in vitro cell culture model was significantly enhanced [76].

#### 2.1.4. Nanomedicines: A Potential Solution to Insulin Administration Challenges

Innovative approaches, such as advanced drug delivery technologies and formulation strategies, are being explored to enhance the bioavailability and therapeutic efficacy of orally administered insulin. These advancements aim to revolutionize diabetes management by providing a more patient-friendly and cost-effective alternative to traditional injection methods [77].

Over the years, the field of nanoscience has witnessed exponential growth, driving promising advancements in the management of diabetes conditions [78]. Nanotechnology, serving as a transformative force in the medical realm, equips researchers with tools to design efficient nano-systems for delivering therapeutic molecules with enhanced benefits. These principles are applied in creating nanomedicines or nanotherapeutics, allowing for the loading of therapeutic agents, thereby improving physicochemical properties and achieving enhanced therapeutic outcomes with precise targeting [79]. Nanotechnology has not only played a pivotal role in the development of cutting-edge glucose monitoring devices but also empowered scientists to create various delivery systems aimed at improving the efficacy of insulin and other antidiabetic molecules in the systemic circulation. These nano-systems offer a superior approach compared to conventional methods, circumventing the harsh metabolic pathways that often reduce the effectiveness of these molecules [80].

Diabetes, especially type 2 with its challenges of insulin resistance or deficiency, presents unique complexities. In a sub-category of type 2 diabetes, a significant number of patients exhibit varying blood glucose levels due to obesity, independent of insulin, while others suffer from insulin deficiency or resistance [81]. Scientists have directed their attention to these diverse diabetic conditions, recognizing the potential of nanomedicines in managing these categories. Recent advancements in nanotechnological approaches have led to innovative delivery systems capable of enhancing the potential of anti-diabetic molecules [82]. Studies emphasize the vital role of various nano-formulations, especially those designed with novel smart polymers, in shielding drug molecules from harsh metabolic pathways and facilitating a controlled release pattern, ensuring sustained levels of insulin in patients [83].

Nanoparticle (NP)-based drug delivery systems (DDSs) have garnered significant attention for their efficacy in transporting therapeutic agents to target cells. Nanocarriers, including liposomes, polymeric NPs, solid lipid NPs (SLNs), chitosan, exosomes, micelles, nanogels, and dendrimers, play a crucial role in entrapping drugs, limiting side effects, and improving bioavailability. These nanocarriers are characterized by biodegradability, biocompatibility, non-toxicity, and the ability to escape the reticuloendothelial system [84].

Insulin encapsulation onto NPs provides protection against enzymatic degradation in the gastrointestinal (GI) tract, thereby improving bioavailability when compared to previous formulations. NPs also enhance insulin permeability across the intestinal mucosa by opening the tight junctions between epithelial cells [84]. Recent developments include long-acting NP formulations containing insulin to reduce the injection frequency and extensive exploration of nanocarriers for oral insulin delivery [85]. Smart nanocarrier-based drug delivery systems, such as glucose-responsive NPs synthesized from dextran, have demonstrated rapid and extended self-regulated insulin delivery, effectively reducing elevated blood glucose levels in mice and minimizing the risk of hypoglycemia [86]. Glucose-responsive self-assembled polyamine nanocarriers have also proven effective in regulating blood glucose concentrations [87]. In the context of oral insulin delivery, nanocarriers enhance transport through both paracellular and transcellular pathways. Chitosan, for instance, enhances intestinal permeability by opening the tight junctions between cells, thereby facilitating paracellular transport. This occurs through interactions of positively charged polymers with the negatively charged cell membrane. The transcellular pathway involves processes such as fusion, endocytosis, and adsorption, with receptor-mediated endocytosis being a major route for insulin-loaded nanocarriers to enter cells [52,88].

## 3. Nanomedicine Technological Advancements

**Figure 3:**
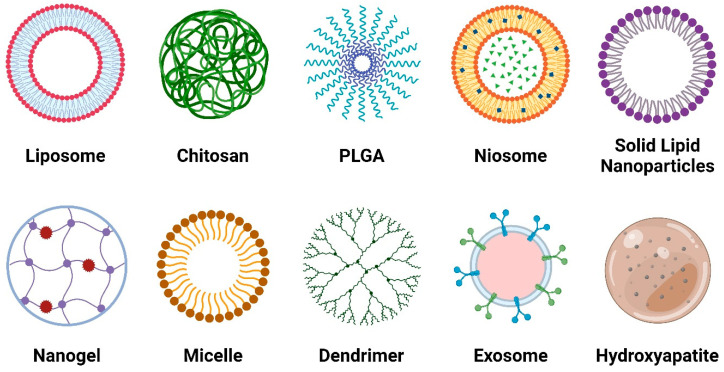
Structural overview of nanocarriers used for insulin delivery.

Traditional injectable insulin has been superseded by novel delivery strategies, which are capable of overcoming well-known delivery limitations. These strategies involve releasing the drug at the target site through diverse carriers employing various mechanisms, following more or less complex kinetics based on the composition of the delivery system. Nanocarriers (Figure 3) have garnered significant attention as the preferred formulations due to their potential to exploit dimensional characteristics for enhanced cellular uptake. Depending on their structural attributes, nanocarriers offer diverse advantages and disadvantages, resulting in a wide array of preparations (Table 1). In the following sections, we provide an overview of different nano-systems and their applications in insulin delivery.

### 3.1. Liposomes

Liposomes, spherical vesicles with lipid bilayers containing an aqueous phase, serve as effective carriers for hydrophilic drugs such as insulin due to their internal structure. This characteristic facilitates the encapsulation of therapeutic agents, thereby offering protection during transportation. The degree of biocompatibility and biodegradability of liposomes varies depending on their composition and synthesis methods [125]. While liposomes typically exhibit particle sizes ranging from a few micrometers to 30 nanometers, polar lipids can also self-assemble into various colloidal particle forms beyond the conventional bilayer shapes, influenced by factors such as the temperature, molecule shape, and environmental conditions. Recent studies have demonstrated the potential of liposomes with specific characteristics, such as a size range of 150–210 nm and negative surface charge, in decreasing blood glucose levels and increasing insulin concentrations in vivo [50]. To overcome challenges like instability in the gastrointestinal tract, modifications such as functionalization with cell-penetrating peptides (CPPs) show promise in improving stability and uptake through endocytosis [50,126]. Additionally, folate-targeted PEGylated liposomes, designed for insulin delivery, demonstrated enhanced bioavailability and cellular uptake through folate receptor-mediated endocytosis [50]. Liposomal chitosan gel, developed for wound healing with encapsulated insulin, exhibited stability and sustained release, showcasing potential clinical benefits [127]. Moreover, sodium-glycodeoxycholate (SGDC)-incorporated elastic liposomes significantly enhanced insulin permeation across buccal tissues [128]. Innovative approaches, such as the development of glucose-sensitive multivesicular liposomes using the double emulsion technique by Liu et al., show promise for self-regulated insulin administration. Liu et al. demonstrated both in vitro and in vivo that these systems would be able to control blood glucose levels within a normal range [129]. Shafiq et al. have explored the potential of using liposomes derived from the fat globule membrane (MFGM) of camel milk as carriers for insulin. In an in vivo study, diabetic rats induced by STZ were treated with these camel milk-derived liposomes. The results revealed substantial reductions in the blood glucose levels, as well as significant decreases in the bilirubin, alkaline phosphatase, albumin, and alanine aminotransferase levels, highlighting the effectiveness of this novel insulin delivery method [130]. Wu et al. tested liposomes loaded with arginine–insulin complexes (AINS) incorporated into a hydrogel prepared from cysteine-modified alginate (Cys-Alg) to form liposome-in-alginate hydrogels (AINS-Lip-Gel). Their ex vivo study demonstrated that the intestinal permeation of AINS and AINS-Lip was approximately 2.0- and 6.0-fold higher, respectively, compared to free insulin. Moreover, their in vivo evaluation showed that the hydrogel effectively retarded the early release of insulin (~30%) from the liposomes and enhanced the intestinal mucosal retention. In vivo experiments further revealed that the AINS-Lip-Gel released insulin in a controlled manner and exhibited strong hypoglycemic effects [131].

### 3.2. Chitosan

Chitosan, a natural polysaccharide renowned for its mucoadhesive properties and facile encapsulation capabilities, is widely recognized as a pivotal material in the formulation of nanocarriers for oral insulin delivery. Chitosan NPs offer further advantages in overcoming the limitations associated with administering insulin alone. These nanocarriers possess a positive surface charge which, in acidic environments like the gastric tract, leads to enhanced protonation, rendering them more cationic [132]. This promotes greater bioavailability as they interact with negatively charged mucus, thereby prolonging the residence time and improving the drug absorption by creating space between the tight junctions among epithelial cells. However, the acidic environment is only characteristic of the stomach. Starting from the small intestine (pH about 6.8), the GI environment becomes closer to alkaline (up to 7.4 in the ileum), which leads to deprotonation of chitosan amino groups and destruction of polyelectrolyte complexes [51,133]. Moreover, the preparation of chitosan NPs is relatively straightforward, involving electrostatic interactions to complex with anionic insulin, minimizing the risk of affecting its structure and integrity. Despite chitosan’s known drawbacks, such as its poor mechanical properties and low antibacterial activity, these challenges can be mitigated by complexing it with other natural or synthetic polymers [134]. Innovation lies in researchers’ ability to combine chitosan with other materials to enhance its desirable properties. For example, folate-conjugated chitosan nanoparticles (NPs) increased insulin stability in the harsh GI tract and improved cellular uptake, resulting in enhanced hypoglycemic activity in vivo [135]. Modification with poly(sodium 4-styrenesulfonate, PSS) improved stability in acidic conditions, while a carboxymethyl-β-cyclodextrin-grafted chitosan NPs formulation exhibited excellent encapsulation efficiency and sustained drug release, significantly reducing the blood glucose levels in mice [136,137], or a thermosensitive copolymer incorporating the chitosan–zinc–insulin complex showcased reduced burst release and improved stability during storage [138].

Studies conducted both in vitro and in vivo have demonstrated that chitosans and their derivatives possess anti-diabetic properties by affecting insulin resistance, glucose uptake, and lipid metabolism [139]. Ju et al. showed that in insulin-resistant rats induced by a high-energy diet combined with streptozocin (STZ), chitosan administration led to reductions in the fasting insulin and blood glucose levels, improved insulin sensitivity, and enhanced oral glucose tolerance [140]. Furthermore, in STZ-induced diabetic mice, chitosan supplementation attenuated damage to pancreatic islets and prevented nuclear pyknosis and atrophy of pancreatic cells [141]. Pang et al. developed an oral insulin delivery system using chitosan (Chi) and alginate (Alg) dual-coated double-layered hydroxide (LDH) nanocomposites (Alg-Chi-LDH@INS). These nanocomposites, with a size of ~350.8 nm and a charge of ~−13.0 mV, effectively mitigated burst insulin release in acidic conditions. Flow cytometry showed enhanced uptake of LDH@INS by Caco-2 cells due to the chitosan coating. The in vivo study on diabetic mice demonstrated significant hypoglycemic effects, reducing the blood glucose levels by ~50% at 4 h, highlighting the system’s potential for diabetes treatment [142]. Abd-Alhussain et al. compared insulin-loaded nanoparticles (NPs) to subcutaneous insulin in diabetic rats. They used biodegradable chitosan-capped NPs with soluble human insulin (20 IU/kg). While the glucose reduction at 6 h was not significant, the insulin levels were significantly higher at 12 and 24 h in the NP-treated rats compared to those administered subcutaneous insulin. The results suggest that chitosan-based NPs can maintain good glycemic control for up to 24 h and are a potential carrier for oral insulin delivery [143]. Maurya et al. developed mannose ligand-conjugated nanoparticles using a quality-by-design approach to enhance oral insulin delivery. They identified critical formulation attributes and process parameters affecting nanoparticle quality. Mannosylated chitosan nanoparticles, prepared by the inotropic gelation method and encapsulated to protect from acidic environments, showed optimal size, charge, and drug entrapment. Cell studies confirmed the safety and selective uptake by M-cells, enhancing insulin bioavailability [144]. Additional recent preclinical studies have highlighted the potential of chitosan-based NPs in enhancing oral insulin delivery. Thiolated chitosan NPs were incorporated into fast-disintegrating dosage forms, showing promising mucoadhesive properties and hypoglycemic effects [145]. Mercaptonicotinic acid-activated thiolated chitosan NPs demonstrated improved mucoadhesivity and insulin permeability [146]. Virus-capsid mimicking NPs with biotin-grafted chitosan exhibited superior transmucosal penetration and hypoglycemic response [147]. Dinitro salicylic acid-functionalized chitosan NPs effectively reduced the serum glucose levels in diabetic rats [148]. A “ternary mutual-assist” system with vitamin B12–chitosan improved insulin stability and absorption [149]. Chitosan derivative-based NPs loaded with fibroblast growth factor-21 (FGF-21) showed durable drug release and biocompatibility [150]. Net-neutral particles formed with chitosan improved mucosal transport and bioavailability [151]. Acetylated cashew gum and chitosan NPs reduced blood glucose without toxicity [152]. Silica-coated chitosan–dextran sulfate NPs provided controlled insulin release [153]. Lastly, chitosan/PEG and albumin-coated NPs maintained insulin bioactivity and stability in simulated gastrointestinal conditions, collectively indicating chitosan’s pivotal role in optimizing oral insulin delivery systems [154].

In a clinical study involving Korean patients aged 20 to 75, daily chitosan supplementation of 1.5 g for three months effectively lowered the postprandial serum glucose levels [155]. Finally, it has been reported that chitosan oligosaccharides may contribute to the protection against oxidative stress [156]. Given the importance of managing and preventing complications associated with diabetes, this property could present another valuable tool [26,103].

### 3.3. PLGA (Poly(lactic-co-glycolic) Acid)

PLGA, renowned for its exceptional biocompatibility and biodegradability, stands out as a widely utilized organic material in the development of nanocarriers tailored for insulin delivery. Lactic and glycolic acid monomers undergo copolymerization to generate PLGA, an aliphatic polymer with a polyester backbone. This copolymerization process allows for the creation of a wide array of formulations by adjusting the ratio of lactic and glycolic acid monomers. By varying this composition, the crystallinity, hydrophilicity, and glass transition temperature of the copolymer can all be controlled. Since lactic acid is more hydrophobic than glycolic acid, a higher ratio of lactic acid results in a more hydrophobic copolymer. Consequently, modifying the composition in this way can decrease the rate at which water penetrates a device [157]. Additionally, PLGA is renowned for its non-toxic nature and is duly approved by both the Food and Drug Administration and the European Medicines Agency for medical applications. Within the body, it is metabolized into lactide and glycolate monomers, which are further transformed into CO_2_ and H_2_O through the Krebs cycle. The rate of drug release from PLGA is determined by its degradation, which can be regulated by the ratio of lactide to glycolide monomers and the molecular weight. Despite the previously demonstrated in vitro cellular uptake of insulin trapped in PLGA NPs through the early and late endosomes pathway, in vivo the effects of the administration of unmodified PLGA alone on blood glucose levels are limited [158]. This limitation is primarily attributed to its poor muco-permeabilization and muco-adhesion capacity, stemming to its hydrophobic and anionic nature, as well as the inability to completely protect insulin in the gastric environment. Consequently, there is a need to coat and/or modify the PLGA. The most prevalent modification involves conjugation to PEG, although the literature is continually expanding with various conjugation strategies. For instance, PLGA, a widely used polymeric NP system, enhances insulin absorption through the transcellular pathway. PLGA/chitosan NPs demonstrated significant hypoglycemic effects [159], while montmorillonite PLGA nanocomposites exhibited increased insulin stability in low-pH environments and sustained release in simulated gastrointestinal fluid [160]. The studies on oral insulin delivery using PLGA-based systems show promising advancements in improving the bioavailability and efficacy. Zhou et al. demonstrated that a “ternary mutual-assist” nano-delivery system using PLGA, ionic liquids, and vitamin B12–chitosan (VB12-CS) enhanced insulin protection and absorption, leading to a significant blood glucose reduction in diabetic mice [149]. Liu et al. reported that zwitterionic materials, although challenging to coat on hydrophobic nanoparticles, were successfully applied using zwitterionic Pluronic analogs, resulting in stable PLGA@PPP4K nanoparticles that effectively lowered the blood glucose levels in diabetic rats [161]. Asal et al. investigated chitosan-based nanoparticles; particularly, they showed that chitosan gold nanoparticles functionalized with PLGA achieved high insulin entrapment and controlled release, significantly reducing the blood glucose levels in diabetic rats [162]. Furthermore, Li et al. studied a novel PLGA-Hyd-PEG nanoplatform that exhibited pH-responsive properties, enhancing mucosal penetration and cellular uptake, and effectively lowering the blood glucose levels in diabetic rats [163].

### 3.4. Niosomes

Niosomes, synthetic microscopic vesicles mainly comprised of non-ionic surfactants and cholesterol as excipients, represent a versatile and promising class of drug delivery vehicles. These nanocarriers exhibit excellent potential in drug delivery systems due to their ability to serve as reservoirs for drugs, leading to sustained and prolonged release profiles while accommodating substances with varying solubilities [124]. Additionally, their inherent biocompatibility and low toxicity make them attractive candidates for therapeutic applications [164]. Niosomes can be categorized into different types based on their sizes and bilayers, including large unilamellar vesicles (LUVs), small unilamellar vesicles (SUVs), and multilamellar vesicles (MLVs). They offer advantages such as enhanced drug entrapment efficiency and controlled release kinetics [165]. Ning et al. investigated niosomes as vaginal delivery systems for insulin, demonstrating promising results. Specifically, they showed significant reductions in the blood glucose levels with sustained hypoglycemic effects [124]. In a study, niosomes made from polyoxyethylene alkyl ethers (Brij™) were developed for insulin encapsulation. The niosomes, particularly those with Brij 92 and cholesterol in a 7:3 molar ratio, protected insulin against enzymatic degradation, showing potential as sustained-release oral dosage forms for insulin delivery [94].

### 3.5. Solid Lipid Nanoparticles

Solid lipid nanoparticles (SLNs) are composed of solid lipids, such as purified triacylglycerols, waxes, or complex mixtures of acylglycerols, along with surfactants forming an aqueous dispersion around a crystalline lipid core [166]. These submicron (50–1000 nm) colloidal carriers offer biodegradability and are advantageous for large-scale production. Their degradation rate is composition-dependent, being inversely proportional to the length of the fatty acid chains of the acylglycerols, thus enabling predictable and regulated drug release. Various SLN formulations for loading insulin have been developed, typically including lecithin and poly-oxyethylene esters of 12-hydroxystearic acid as surfactants, along with solid lipids like cetyl palmitate, glyceryl monostearate, or glyceryl palmitostearate [167]. VB12-coated gel-core SLNs demonstrated prolonged hypoglycemic effects in diabetic rats by preventing burst release in low-pH environments [108]. Additionally, cationic SLNs protected insulin from enzymatic activity, ensuring stability and sustained release in the media [168]. Muntoni et al. developed SLNs loaded with glargine insulin, which were formulated into solid oral dosage forms to enhance stability and prolong shelf-life. Evaluation in both healthy and diabetic rat models demonstrated significant hypoglycemic effects, particularly with the capsule formulation, suggesting their promise as effective agents for lowering blood glucose levels. However, further optimization is required to refine these SLNs for practical use as orally active insulin preparations [169]. Meanwhile, Zheng et al. focused on SLNs loaded with insulin (INS-SLNs), prepared using a methanol–chloroform mixed solvent system. These nanoparticles exhibited desirable properties such as a small size, excellent stability, and sustained release in simulated intestinal conditions. Cellular studies indicated enhanced uptake and improved transcytosis across intestinal epithelial cells compared to free insulin, highlighting their potential to enhance the delivery of peptide and protein drugs via the oral route [170].

### 3.6. Nanogels

Nanogels are hydrogels at the nanometer scale, ranging from a few nanometers up to 300 nm. They consist of a network of polymer chains containing basic or acidic groups, allowing them to absorb and retain large amounts of aqueous solution. The primary characteristic of nanogels is their swelling capacity, maintained through crosslinks between polymers, which can be either homopolymers or copolymers. Nanogels come in various types: synthetic, natural, and hybrid, as well as neutral, anionic, or cationic, depending on their surface charge. They can internally load biological cargo, facilitated by electrostatic interactions, and externally conjugate different molecules via functional group bonds [171]. Unlike other nanoparticles, nanogels can be structured to respond to specific changes in their environment. Dextran-crosslinked glucose-responsive nanogels demonstrated high encapsulation efficiency and glucose-dependent insulin release [172], while pH- and temperature-sensitive HPMC nanogels provided controlled release, and a multi-responsive nanogel adjusted the release rate based on the glucose concentration [172,173,174].

Baloch et al. explored a novel approach for oral insulin delivery using an insulin-intercalated graphene oxide (In@GO NgC) nanogel composite. Their study investigated the release profile of insulin from In@GO NgC in simulated gastric and intestinal fluids, demonstrating enhanced release in intestinal conditions and stability against enzymatic degradation over 6 h. Comparative analysis with non-intercalated GO nanogels and nanogels without GO underscored the superior release profile and enzymatic stability of In@GO NgC, highlighting its potential for effective oral insulin delivery [174]. Mudassir et al. evaluated pH-sensitive methyl methacrylate/itaconic acid (MMA/IA) nanogels as carriers for oral insulin delivery. Their optimized formulations exhibited a favorable particle size, zeta potential, and high entrapment efficiency crucial for insulin stability and release characteristics in simulated gastrointestinal fluids. The study demonstrated that lyophilization with trehalose preserved the stability of these nanogels, maintaining the insulin integrity. In vivo studies in diabetic rats showed significant reductions in the blood glucose levels following oral administration of the optimized nanogel formulation, indicating their potential as effective carriers for enhancing oral insulin absorption and therapeutic efficacy [172]. Wang et al. synthesized hydroxyethyl methacrylate (HEMA) nanogels via emulsion polymerization and investigated their potential as oral insulin delivery systems. Their characterization studies highlighted the morphology, stability, and enhanced circulation half-life of HEMA nanogels, with minimal uptake by macrophage cells. In vivo studies demonstrated that HEMA nanogels facilitated efficient intestinal absorption of encapsulated insulin, sustaining blood glucose control for up to 12 h with improved bioavailability compared to free insulin. These findings suggest that HEMA nanogels could serve as promising alternatives for oral insulin delivery, offering potential benefits in managing diabetes without the need for injections [175]. Chou et al. developed an injectable insulin-loaded gel composed of self-assembled nanoparticles from carboxymethyl-hexanoyl chitosan (CHC) integrated with lysozyme for sustained basal insulin delivery. In vitro evaluations confirmed the controlled biodegradation and insulin release kinetics of the CHC-lysozyme gel, demonstrating the cytocompatibility of the degradation products. In vivo studies in diabetic mouse models revealed that a single injection of the gel maintained the fasting blood glucose levels within normal ranges for up to 10 days. This injectable system shows promise as a novel long-acting insulin delivery method, potentially improving treatment adherence and reducing the complications associated with conventional insulin therapies [176].

### 3.7. Micelles

Polymeric micelles serve as widely used nanocarriers owing to their inherent properties. In an aqueous milieu, amphiphilic polymers spontaneously organize into a distinct conformation. The resultant structure comprises a hydrophilic outer region and a hydrophobic core, offering potential as a reservoir for sustained drug release. While encapsulating hydrophilic insulin presents complexities, numerous techniques have been developed over time to enable efficient loading. Generally, polymeric complex micelles have demonstrated enhanced insulin-loading efficiency and stability under physiological conditions [177,178,179]. Moreover, innovative delivery strategies have significantly improved insulin delivery. Notably, Liu et al. showcased that glucose and H_2_O_2_ dual-responsive polymeric micelles exhibited considerable hypoglycemic effects in vivo while maintaining good biocompatibility [180]. Bahman et al. designed a poly-(styrene-co-maleic acid) (SMA) micellar system for oral insulin delivery, addressing challenges such as rapid insulin degradation in the stomach and enhancing intestinal absorption. Insulin was encapsulated into SMA micelles in a pH-dependent manner, characterized using dynamic light scattering and HPLC. In vitro studies with Caco-2 cells and ex vivo rat intestinal sections, along with in vivo experiments in diabetic mice, demonstrated effective stimulation of glucose uptake by SMA-insulin. The negatively charged micelles, with a mean diameter of 179.7 nm, showed promising hypoglycemic effects lasting up to 3 h post-administration. Overall, SMA micelles offer a promising strategy for oral insulin delivery, potentially improving diabetes management [181]. Han et al. focused on overcoming the barriers to oral protein drug delivery by developing a zwitterionic micelle platform. These micelles feature a virus-mimetic zwitterionic surface and a betaine side chain, with an ultralow critical micelle concentration, enabling drug penetration through intestinal mucus and enhancing epithelial absorption via transporters. An oral insulin prototype, encapsulated in enteric-coated capsules, exhibited high oral bioavailability (>40%) and customizable insulin action profiles. This biocompatible formulation promises long-term safety and represents a significant advance in oral protein drug delivery, potentially transforming treatment for patients requiring regular injections [182]. Italiya et al. described the development of an orally active nanoformulation for lisofylline (LSF). LSF was encapsulated as its ester prodrug (LSF-linoleic acid (LA) prodrug) into biodegradable polymeric micelles (LSF-LA PLMs), enhancing its pharmacokinetic profile, with significantly improved oral bioavailability (74.86%) compared to free LSF. In vitro studies confirmed the formulation’s stability and efficacy in insulin-secreting cells. In vivo experiments in STZ-induced diabetic rats demonstrated reduced fasting glucose levels and increased insulin levels via both oral and intraperitoneal routes. Additionally, the LSF-LA PLM formulation mitigated inflammation and preserved pancreatic integrity in diabetic animals, suggesting its potential as a therapeutic strategy in type 1 diabetes [183]. Hu et al. explored amphiphilic pH-sensitive block copolymer poly(methyl methacrylate-co-methacrylic acid)-b-poly(2-amino ethyl methacrylate) (P(MMA-co-MAA)-b-PAEMA) as carriers for oral insulin delivery. Synthesized via ARGET ATRP, these copolymers self-assembled into pH-responsive cationic polymeric micelles (PCPMs), characterized by 1H-NMR, FT-IR, and GPC. The PCPMs exhibited pH-sensitive behavior, maintaining stability in acidic environments and increasing in size at a neutral pH. Insulin was efficiently loaded into the PCPMs with low toxicity and a controlled pH-triggered release profile, demonstrating their potential as effective carriers for oral insulin delivery [184].

### 3.8. Dendrimers

Dendrimers are synthetic polymers synthesized by reacting a diamine with methyl acrylate. Their generation can proceed in both divergent and convergent manners, resulting in a macromolecule composed of a core, branching units, and surface functional groups that dictate their properties. Their highly regular architecture facilitates effective surface functionalization with molecules tailored to achieve specific properties [185,186]. For instance, modification with PEG enhances stabilization, preventing macrophage attack [187]. Another example is the caproyl-modified G2 PAMAM dendrimer, which efficiently increased pulmonary insulin absorption through the paracellular and transcellular pathways [188]. Amphiphilic dendrimers based on multi-armed poly(ethylene glycol) (PEG) showed stability under an acidic pH and reduced blood glucose levels [189]. Xian et al. developed a nanoscale complex for autonomous insulin therapy responsive to glucose levels, aiming to enhance diabetes management. Their approach combined a synthetic dendrimer carrier with an insulin analog modified using a high-affinity glucose-binding motif, employing electrostatic and dynamic-covalent interactions. This resulted in an injectable insulin depot capable of providing both glucose-directed and long-lasting insulin availability. The nanocomplex, administered via a single injection, maintained controlled blood glucose levels for at least one week in diabetic swine subjected to daily oral glucose challenges. The serum insulin concentrations increased correspondingly with the elevated blood glucose levels, a notable achievement in glucose-responsive insulin therapy [190].

### 3.9. Exosomes

Exosomes are membrane vesicles composed of specific proteins with vesicular fusion and fission functions, along with lipids. Ranging in size from 30 to 150 nm, exosomes play a physiological role in interacting with target cells and serving as cargo delivery agents within them. Notably, exosomes derived from the pancreas of patients with type 2 diabetes have been found to influence the survival and apoptosis of pancreatic β cells through the miRNAs they transport [191]. Exosomes hold potential as insulin delivery systems. Cell-derived exosomes encapsulated with insulin demonstrated enhanced transport and metabolism of glucose in cells, suggesting their value as nanocarriers for diabetic treatment [192]. Morales et al. achieved successful encapsulation of insulin into exosomes using an electroporation technique. Specifically, they mixed insulin with exosomes and subjected them to electroporation with parameters set at 200 V and 50 μF, followed by an incubation period of 1 h at 37 °C. They noted that the loading efficiency varied among exosomes derived from different cell sources, with optimal efficiency observed under these specified electroporation conditions. Importantly, this method exhibited significantly higher loading efficiency compared to conventional room temperature incubation methods for loading exosomes [189]. Treatment with exosomes isolated from plasma improved the glucose tolerance, insulin sensitivity, and reduced plasma triglyceride levels in mice [193]. Wu et al. investigated the potential of milk-derived exosomes as oral drug delivery vehicles, focusing on insulin encapsulation (EXO@INS) and their effects in type I diabetic rats. They found that EXO@INS exhibited a significantly enhanced and sustained hypoglycemic effect compared to subcutaneously injected insulin. Mechanistic studies revealed that milk-derived exosomes possess active multi-targeting uptake mechanisms, adapt to pH changes during gastrointestinal transit, activate ERK1/2 and p38 MAPK signaling pathways related to nutrient assimilation, and penetrate intestinal mucus effectively [194].

### 3.10. Hydroxyapatite (HAP)

Hydroxyapatite is a highly porous material, making it an excellent candidate material for drug transport, particularly in the development of nanocarriers for insulin delivery. Additionally, it has been shown to be biocompatible and bioactive. Consequently, hydroxyapatite nanoparticles (Ca_10_(PO_4_)_6_(OH)_2_) serve as a foundation for delivery systems aimed at insulin release [84,114]. PEG-functionalized HAP demonstrated effectiveness in this system [195]. Insulin encapsulated into the HAP crystal lattice showcased constant release, regulating the blood glucose levels in rats [77]. Moreover, Zhang et al. developed nano HAP nanoparticles for oral insulin delivery by surface-wrapping them with PEG and conjugating insulin (INS) and gallic acid (GA) with PEG. This innovative approach aimed to improve on traditional nanoparticle carriers by enhancing the hydrophilicity and stability in the gastrointestinal tract, addressing toxicity and biocompatibility concerns. In vivo studies in rat small intestines showed absorption of HAP-PEG-GA-INS by the epithelium, resulting in reduced blood glucose levels in type 1 diabetes rats after intragastric administration [195]. Scudeller et al. investigated insulin-loaded calcium phosphate nanoparticles (HA, SrHA, ZnHA) for oral diabetes treatment and bone cell stimulation. They found that insulin adsorption was influenced by electrostatic forces, with ZnHA enhancing adsorption and SrHA inhibiting it due to surface changes. Circular dichroism revealed insulin conformational changes, particularly pronounced on ZnHA. SrHA-loaded insulin improved cell proliferation in vitro, while HA and ZnHA had minimal effects [196].

## 4. Safety Considerations

The safety concerns associated with nanomedicine, including the potential toxicity, immunogenicity, and long-term effects on physiological systems, must still be carefully addressed to ensure patient safety [197]. Nanoparticles (NPs) can elicit toxicity due to their small size and high surface area, which may lead to interactions with biological molecules and cellular structures. Additionally, certain nanomaterials may provoke an immune response, causing immunogenicity issues that could compromise treatment efficacy. The long-term effects on physiological systems remain a concern, as the biodistribution and accumulation of NPs in organs over time are not yet fully understood [197]. To mitigate these risks, the FDA has recently issued guidance aimed at promoting the safe development of nanotechnology-based products for clinical use [198]. These guidelines emphasize the importance of extensive characterization of nanomaterials, understanding their intended applications, including the stability and release mechanisms, and assessing how these attributes impact product quality, safety, and efficacy, especially concerning routes of administration like insulin delivery. Additionally, the breakdown, elimination, and biodegradation of nanomaterials, initially evaluated through animal studies, are critical aspects. Furthermore, rigorous characterization of excipients is essential, given their potential impact on nano-level drug absorbency [198]. Current nanomedicine research increasingly aligns with essential safety criteria by focusing on the rigorous characterization of nanomaterials and comprehensive assessment of their applications, and by conducting thorough preclinical and clinical assessments, potential adverse effects can be identified and managed effectively, ensuring the safety and efficacy of nanomedicine formulations for diabetes management and other therapeutic applications [199].

## 5. Future Directions

In the field of nanomedicine for precision insulin delivery, future directions hold promise for transformative advancements aimed at addressing current limitations and optimizing therapeutic outcomes. These include challenges such as achieving sufficient oral bioavailability due to enzymatic degradation in the gastrointestinal tract and poor absorption across the intestinal epithelium, which limits the effectiveness of oral insulin formulations as a non-invasive alternative to injections. The immunogenicity of nanomaterials used in insulin delivery systems is another significant concern, potentially compromising the therapeutic benefits. Additionally, scaling up production and ensuring consistency in nanoparticle synthesis pose challenges, with variability in the size, shape, and surface properties impacting performance and safety. Researchers are exploring solutions such as protective coatings or modifications to shield insulin from degradation, surface modifications to reduce immunogenicity, and advanced manufacturing techniques to ensure uniform nanoparticle production [200]. A primary focus involves the development of next-generation nanocarriers with enhanced targeting capabilities, allowing for precise delivery of insulin to specific tissues or cells associated with diabetes pathophysiology. This includes exploring stimuli-responsive nanomaterials that can selectively release insulin in response to physiological cues, such as glucose levels or pH variations [201]. Farokhzad et al. are working on a novel approach to overcoming the gastrointestinal barriers for oral delivery of biologics by targeting nanoparticles to the FcRn in the intestines. Using insulin as a model, they aim to develop FcRn-targeted, insulin-encapsulated nanoparticles that can efficiently cross the intestinal epithelium via transcytosis, enhancing bioavailability (project number: 5R01EB015419-02) [202]. On the other hand, Li et al. aim to develop a fast-acting oral insulin formulation using milk protein casein-coated nanoparticles (casNPs) to encapsulate insulin and the absorption enhancer sodium caprate (C10). This innovative approach targets the small intestine, protecting insulin from gastric degradation and enabling enzyme-triggered release for improved bioavailability. The study involves optimizing the casNP/insulin/C10 formulation, tracking delivery and release in diabetic mice, and evaluating its efficacy in controlling hyperglycemia (project number: 1R41DK131761-01) [203]. Furthermore, Majeti et al. aim at developing an effective oral insulin delivery system using ligand-directed nanoparticles (project number: 5R01DK125372-04). This project focuses on optimizing nanoparticle chemistry to enhance insulin stability and absorption in the gastrointestinal tract. The study will evaluate the drug disposition and therapeutic outcomes under various physiological and pathological conditions, establishing a robust method for early insulin therapy in type 2 diabetic patients, potentially overcoming the drawbacks of current insulin injections and improving patient compliance and management [204].

Integrating advanced imaging modalities and biosensors into nanomedicine platforms enables real-time monitoring of the insulin distribution and therapeutic efficacy, facilitating personalized treatment strategies. This real-time feedback can significantly improve the management of insulin levels, reducing the risk of hypoglycemia and other complications [205]. The convergence of nanotechnology with emerging fields such as gene editing and regenerative medicine also holds revolutionary potential for diabetes management. This includes engineering insulin-producing cells or tissues for transplantation, offering potential long-term solutions for insulin dependence. Moreover, there is potential in developing hybrid systems that combine nanocarriers with smart devices or wearable technology, providing continuous monitoring and automated insulin delivery systems that adjust to the user’s needs dynamically [205,206]. Furthermore, in recent years, glucose-responsive insulin release nano-systems have become a significant focus of research [103]. These systems leverage glucose-sensitive elements such as glucose oxidase, concanavalin A, and phenylboronic acid to release insulin in response to hyperglycemic conditions, while minimizing or preventing insulin release under normal glucose levels [207,208,209]. This “smart” system primarily targets subcutaneous injection, with limited exploration in oral insulin delivery systems [210]. The “smart” glucose-responsive mechanism prevents insulin overdosage and reduces the risk of hypoglycemia. If successfully adapted for oral administration, this system could better mimic endogenous insulin secretion and distribution, thereby improving blood glucose level regulation and control, especially if incorporated with a hybrid system for automated insulin delivery [211,212]. Gu Zhen et al. are working on the development of a glucose-responsive insulin delivery system using glucose derivative-modified insulin and glucose transporters on red blood cells (project number: 7R01DK112939-02). The system will include polymeric and liposomal nanoparticles, integrated into a painless microneedle-array patch for precise blood glucose control [213]. Similarly, Li et al. propose an innovative glucose-responsive insulin delivery system that mimics pancreatic β-cells to improve diabetes care (project number: 5R01DK112939-06). Their system uses Glu-insulin interacting with GLUTs on red blood cells to release insulin during hyperglycemia. Li et al. will develop two formulations: polymeric nanoparticles coated with red blood cell membranes and liposomal nanoparticles integrated with glucose transporters. These will be incorporated into a “smart insulin patch” for up to 48 h of regulation. Their study will optimize the glucose responsiveness, effectiveness, and biocompatibility in diabetic mouse and rat models, aiming to revolutionize insulin-dependent diabetes therapy [214]. Collaborative efforts between multidisciplinary research teams, alongside sustained investment in preclinical and clinical studies, will be crucial in driving these future directions toward clinical translation. Such efforts are essential to ultimately improving outcomes for individuals living with diabetes by delivering more effective, personalized, and less invasive treatments.

## 6. Conclusions

In today’s pharmaceutical landscape, drug delivery systems based on nanoparticles (NPs) play a crucial role. Nanocarriers are utilized to load drugs, aiming to mitigate their adverse effects while enhancing their bioavailability and efficacy. Particularly, in recent years, there has been significant interest in developing insulin delivery systems for the treatment of diabetes. NPs have demonstrated the ability to enhance insulin permeability across the intestinal mucosa by opening the tight junctions between epithelial cells. Looking ahead, it is conceivable that nanocarrier-based insulin delivery systems may eventually replace conventional methods such as subcutaneous insulin injections in the treatment of diabetic patients.

## Figures and Tables

**Table 1:** Systems for delivering insulin nanoparticulates to treat diabetes mellitus.

Nanocarriers	Administration Route	Effects In Vivo	Challenges
Liposomes	Oral	- Increased hypoglycemic effect [89] - Improved insulin absorption and oral bioavailability [90] - Conquer mucus and epithelium barriers [91] - Increased retention time in lungs, reducing extra pulmonary side effects [91] - Improved proteolytic stability in oral administration [66] - Chemical responsive release [92] - Sustained release and transmucosal delivery [93]	- Non-uniform coating [66] - Lower entrapment efficiency compared to polymeric carriers [94] - Leakage due to instability, especially autoxidation of cholesterol [94] - Possible allergic reactions due to lipid presence [95,96]
Chitosan	Oral/Nasal/ Transdermal	- Overcome mucus and epithelium barriers [97] - Increased bioavailability [98] - 54.19% reduction in blood sugar level after 4 h [99] - Normal histological findings, no signs of inflammation or ulceration [99]	- Insulin-loading efficiency in chitosan nano-formulations [100] - Ensuring stability and controlled release of insulin [100] - Better understanding of chitosan nanoparticles’ interactions with biological barriers [101]
PLGA	Oral	- Reduction of blood sugar level [102] - Prolonged hypoglycemic effect [102]	- Potential lack selectivity in interacting with mucosal surfaces [103]
SLNs	Oral	- Reduction of blood sugar level [43]	- Low oral bioavailability due to stomach degradation [43] - Inactivation and digestion by proteases in the luminal cavity [43] - High molecular weight and lack of lipophilicity, leading to reduced intestinal absorption [104]
Nanogels	Oral	- Improved hypoglycemic effects [105] - Increased bioavailability [105]	- Specific size and stability [105] - Precise controlled release [106] - Tissue penetration [107] - Biocompatibility and immunogenicity [108]
Micelles	Oral	- Prevention of insulin aggregation [109] - Increased bioavailability [109]	- Limited payload capacity [110] - Stability issues [111] - Biocompatibility concerns [112] - Controlled release challenges [113]
HAP	Oral	- Regulation of blood glucose levels in rats by continuous insulin release [114]	- Low insulin-loading capacity [115] - Limited bioadhesiveness [116]
Dextran nanoparticles	Subcutaneous	- Prolonged hypoglycemic effect [117]	- Biodegradation and clearance [118] - Possible immunogenicity [119]
Polyethylene glycol (PEG) nanoparticles	Oral	- Enhanced hypoglycemic effects [120] - Improved bioavailability [120]	- Possible allergic reactions [121] - Controlled release and targeting efficiency [120]
Hydrogels	Oral/Subcutaneous	- Targeted delivery [122] - Enhanced bioavailability [122]	- Possible adverse immune responses or tissue reactions [123] - Insulin stability [123] - Precise control over insulin release kinetics to match physiological requirement [123]
Niosomes	Oral/Mucosal	- Stabilized against enzymatic degradation in oral and vaginal delivery [124] - Prolonged bioactivity for 6 h [124]	- Low entrapment efficiency [94] - Instability due to alteration in molecular arrangement of surfactants [94]

## Data Availability

No dataset was generated for the publication of this article.
